# Expression of alternatively spliced variants of the Dclk1 gene is regulated by psychotropic drugs

**DOI:** 10.1186/s12868-018-0458-4

**Published:** 2018-09-12

**Authors:** Magdalena Zygmunt, Dżesika Hoinkis, Jacek Hajto, Marcin Piechota, Bożena Skupień-Rabian, Urszula Jankowska, Sylwia Kędracka-Krok, Jan Rodriguez Parkitna, Michał Korostyński

**Affiliations:** 1 Department of Molecular Neuropharmacology, Institute of Pharmacology of the Polish Academy of Sciences, Smetna 12, 31-343 Krakow, Poland; 2 Laboratory of Proteomics and Mass Spectrometry, Malopolska Centre of Biotechnology, Jagiellonian University, Krakow, Poland; 3 Department of Physical Biochemistry, Faculty of Biochemistry, Biophysics and Biotechnology, Jagiellonian University, Krakow, Poland

**Keywords:** Dclk1, Alternative transcription, Psychotropic drugs, Nucleus accumbens, Prefrontal cortex

## Abstract

**Background:**

The long-term effects of psychotropic drugs are associated with the reversal of disease-related alterations through the reorganization and normalization of neuronal connections. Molecular factors that trigger drug-induced brain plasticity remain only partly understood. Doublecortin-like kinase 1 (*Dclk1*) possesses microtubule-polymerizing activity during synaptic plasticity and neurogenesis. However, the *Dclk1* gene shows a complex profile of transcriptional regulation, with two alternative promoters and exon splicing patterns that suggest the expression of multiple isoforms with different kinase activities.

**Results:**

Here, we applied next-generation sequencing to analyze changes in the expression of *Dclk1* gene isoforms in the brain in response to several psychoactive drugs with diverse pharmacological mechanisms of action. We used bioinformatics tools to define the range and levels of *Dclk1* transcriptional regulation in the mouse nucleus accumbens and prefrontal cortex. We also sought to investigate the presence of DCLK1-derived peptides using mass spectrometry. We detected 15 transcripts expressed from the *Dclk1* locus (FPKM > 1), including 2 drug-regulated variants (fold change > 2). Drugs that act on serotonin receptors (5-HT2A/C) regulate a subset of *Dclk1* isoforms in a brain-region-specific manner. The strongest influence was observed for the mianserin-induced expression of an isoform with intron retention. The drug-activated expression of novel alternative *Dclk1* isoforms was validated using qPCR. The drug-regulated isoform contains genetic variants of DCLK1 that have been previously associated with schizophrenia and hyperactivity disorder in humans. We identified a short peptide that might originate from the novel DCLK1 protein product. Moreover, protein domains encoded by the regulated variant indicate their potential involvement in the negative regulation of the canonical DCLK1 protein.

**Conclusions:**

In summary, we identified novel isoforms of the neuroplasticity-related gene *Dclk1* that are expressed in the brain in response to psychotropic drug treatments.

**Electronic supplementary material:**

The online version of this article (10.1186/s12868-018-0458-4) contains supplementary material, which is available to authorized users.

## Background

Psychiatric disorders are associated with complex patterns of abnormal neuronal activity and maladaptive plasticity [1]. Treatment with psychotropic drugs aims to restore the normal function of the affected circuits; long-term therapeutic benefits may depend on the ability of drugs to restore normal plasticity. Consistent with this notion, commonly used psychotropic drugs robustly induce molecular effects on the brain associated with neuroplastic alterations [2, 3].

The dynamic process of cytoskeletal reorganization is one of the key components of brain plasticity [4]. Based on findings from rodent studies, the canonical *Dclk1* transcript encodes a protein that mediates microtubule polymerization and is involved in neurogenesis and neuronal plasticity by regulating dendritic outgrowth and synapse formation [5]. This gene is widely expressed in the nervous system and has been reported to show persistent expression in adult neurons [6]. Moreover, genetic variants of *DCLK1* are associated with psychiatric disorders in humans [7, 8]. These observations suggest a potential role for *Dclk1* in the formation of drug-inducible changes in the brain.

The *Dclk1* gene consists of 20 exons that undergo alternative splicing, resulting in several known variants [6, 9, 10]. Previously identified *Dclk1* transcripts include both mRNAs coding for distinct proteins and noncoding, regulatory RNAs. The multiple isoforms of this gene are differentially expressed and have different kinase activities [6, 11]. The isoforms are classified into four groups expressed from the following two distinct promoters: the full-length variant (containing both DCX and kinase domains), a kinase-lacking isoform (DCL), a doublecortin-lacking isoform (CPG16) and the CaMK-related peptide (CARP/Ania-4) lacking both DCX and kinase domains. The mechanism involved in transcriptional regulation of the diverse alternatively spliced variants of *Dclk1* remains elusive. The importance of the expression of alternatively spliced gene products from a single gene locus in brain physiology and disease progression has been reported [10, 12]. Therefore, acquiring gene expression profiles from all gene variants is a necessary first step in understanding the specific functions of proteins and identification of disease-relevant isoforms.

Here, we investigate drug-induced alternative transcription from the *Dclk1* locus. We used next-generation sequencing to map all transcripts of the *Dclk1* gene, both currently annotated in the mouse genome as well as putative novel RNAs. We found that the gene expression patterns are both drug- and brain-region-specific. Moreover, we identified a new *Dclk1* variant that is specifically regulated by psychotropic drugs acting on the serotonin system.

## Materials and methods

### Animals

Adult male (8–10 weeks old) C57BL/6N mice (Charles River Laboratories, Wilmington, Massachusetts, USA) were housed in Plexiglas cages (Type II L, 2–5 animals per cage) containing Aspen Laboratory bedding (MIDI LTE E-002, Abedd) in a conventional facility on a 12 h light/dark cycle with ad libitum access to water and chow (RM1 A (P), Special Diets Services) and an ambient temperature of 22 ± 2 °C. All the experiments were conducted in accordance with the European Union guidelines for the care and use of laboratory animals (2010/63/EU). Experimental protocols were reviewed and approved by the 2nd Local Institutional Animal Care and Use Committee (IACUC), Institute of Pharmacology Polish Academy of Sciences in Kraków (permit number: 1156/2015).

### Drug treatment

Mice received a single intraperitoneal injection (vol. 10 ml/kg) of haloperidol (1 mg/kg), risperidone (0.5 mg/kg), methamphetamine (2 mg/kg), venlafaxine (16 mg/kg), mianserin (20 mg/kg) or ketamine (20 mg/kg) dissolved in saline, or saline with a drop of 0.1 M HCl in the case of haloperidol and risperidone. Regarding peptide analyses, mice were injected with mianserin (20 mg/kg) daily over 5 days. All animals were decapitated 2 h after treatment, and brains were then extracted and dissected with needles under a binocular. Drug doses that produce robust and comparable gene expression alterations were selected based on our previous experience [2, 3, 13]. Doses were selected to provide a reasonable comparison of the effects of each drug on the molecular level. All of the drugs were shown to cross the blood–brain barrier [14] and accumulate in the brain within minutes of administration [15–18].

### Tissue collection and RNA isolation

Tissue extraction was performed as described previously [3, 13]. Briefly, whole brains were incubated in RNAlater reagent (Ambion) overnight and then coronally sectioned into 125 μm slices using a Vibratome (Leica). The prefrontal cortex (PFCx) and nucleus accumbens (NAc) were dissected with needles under a binocular microscope with a Paxinos atlas as a Ref. [19]. The cingulate, prelimbic, infralimbic and part of the dorsal peduncular cortex were collected from the area approximately + 1.90 to + 1.15 mm from the bregma. The shapes of the corpus callosum and anterior commissure were used to assess the distance from the bregma. Tissue samples were placed in RNAlater reagent and preserved at − 70 °C. The samples were homogenized in 1 ml of TRIzol reagent (Invitrogen, Carlsbad, CA, USA). RNA was isolated according to the manufacturer’s protocol and was further purified using the RNeasy Mini Kit (Qiagen Inc.). The RNA quality was determined using an Agilent 2100 Bioanalyzer (Agilent, Palo Alto, CA, USA).

### Microarray data analysis

In this study, we reanalyzed our previously published microarray data for striatal gene expression profiles produced by 18 major psychoactive drugs at 1, 2, 4 and 8 h after acute administration (Please see: [2] for details). Briefly, the analysis and quality control of 324 microarrays were performed using the BeadArray R package v1.10.0. After background subtraction, data were normalized using quantile normalization and then log_2_-transformed. The results were standardized to reduce the effects of hybridization batches using z-score transformation. Genes2mind was used to visualize the results (http://genes2mind.org) [20].

### Whole-transcriptome sequencing

The procedure was performed as described previously [3, 13]. Total RNA (1 μg) was ribo-depleted using the RiboMinus Eukaryote Kit v2 (Ambion). rRNA-depleted RNA was used to prepare the RNA-seq library generated using the Ion Total RNA-seq Kit v2. Templates were prepared using emulsion PCR (ePCR) with the Ion OneTouch™ 2 Instrument and the Ion PI™ Template OT2 200 Kit v3. Sequencing was performed using an Ion PI™ Sequencing 200 Kit v3 and the Ion PI™ Chip v2 (Life Technologies). The template-positive ion sphere particles (ISPs) were loaded onto an Ion PITM Chip v2 and sequenced (single end reads > 100 bp).

### NGS data analysis

The quality of the NGS data was assessed using FastQC. The RNA-seq reads were aligned using TopHat 2.0.1 followed by Tmap 3.0.2. The transcript FPKM (Fragments Per Kilobase of transcript per Million fragments mapped) levels were quantified using Cufflinks v2.2.1 and GTF from the Ensembl gene database. Statistical significance was tested using ANOVA on log_2_(1 + x) values [3]. The false discovery rate (FDR) was estimated using the Benjamini–Hochberg method. All statistical analyses were performed using R software v3.3.1. Transcript annotation and classification were performed using the BioMart interface to the Ensembl database. Identification of transcription factor binding sites in the promoter regions corresponding to the identified transcripts was performed using the seqinspector (seqinspector.cremag.org) [20]. The data stored in seqinspector included ENCODE ChIP-seq tracks and data deposited in the Gene Expression Omnibus (GEO).

### Variant identification

Initial analysis of transcripts from the *Dclk1* locus was based on GRCm38.p5/mm10, which lists 15 variants. A new variant was identified in the mouse NAc after mianserin treatment using Cufflinks. The new transcript was selected based on the abundance level (FPKM) calculated by Cufflinks; selection criteria were F (min-isoform-fraction) = 0.005 and j (pre-mRNA-fraction) = 0.15. The depth parameter for each nucleotide of the *Dclk1* transcript was computed using Samtools depth v0.1.19. The abundance levels for intron regions were calculated as the median read coverage of the intron.

### Quantitative PCR

Reverse transcription was performed with the Omniscript Reverse Transcriptase (Qiagen Inc.). qPCR was performed using TaqMan Gene Expression Assays (“probe 1”: Mm00444950_m1—exons 4–5 of *Dclk1*; “probe 2”: Mm01545304_m1—exons 6–7 of *Dcl*; “probe 5”: Mm01512375_m1—exons 13–14 of *Cpg16*; “probe 3”: custom designed for 5′ intron 6 of *Dclk1* using Custom TaqMan Assay Design Tool, assay ID: AJ20T2J, “probe 4”: custom designed for 3′ intron 6 of *Dclk1*, assay ID: AI0IY5 V). The reactions were run on the CFX96 Real-Time system (Bio-Rad). Each template was generated from an individual animal. Expression of the hypoxanthine–guanine phosphoribosyltransferase 1 (*Hprt1*) transcript was used to control for variations in cDNA concentrations. The abundance of each RNA was calculated as 2^−(threshold cycle)^. The data were analyzed using one-way analysis of variance (ANOVA) followed by Tukey’s HSD.

### Protein isolation

For the proteomics assessment, NAc samples were collected after 5 days of mianserin treatment. Immediately after removal, the tissue was homogenized in 1% SDS using the Rotor Stator Homogenizer (IKA^®^-Werke, Staufen, Germany) and cleared by centrifugation (16,000*g* for 3 min). The protein concentration in the supernatant was determined using the BCA Protein Assay Kit (Sigma-Aldrich). Samples containing 50 μg of protein were heated in Laemmli 6 × loading buffer for 5 min at 95 °C and resolved by sodium dodecyl sulphate–polyacrylamide gel electrophoresis (SDS-PAGE; 18% Criterion™ TGX™ Precast Gels, Bio-Rad). After electrophoresis, the gel was transferred to 40% methanol/20% acetic acid and stained with Coomassie brilliant blue R250 overnight. Bands corresponding to the < 10 kD fraction of peptides according to the Polypeptide 1.4–26.6 kD SDS-PAGE Standard (Bio-Rad, #1610326) were excised from the gel.

### LC–MS/MS analysis

The procedure is based on a previously reported protocol [21]. First, we injected the heavy-labeled peptide for shotgun LC–MS/MS analysis at 1 pmol. Peptide retention time was determined and a spectral library was constructed in Skyline software version 3.7. Then, the peptide was measured using the scheduled parallel reaction monitoring (PRM) mode. At 10 fmol per injection no contamination with light counterparts was observed.

The gel band was alternately washed with 25% acetonitrile (ACN)/25 mM (NH_4_)HCO_3_ (ABC) and 50% ACN/25 mM ABC, dehydrated with 100% ACN and then air dried. Then, the band was re-swelled in 25 mM ABC containing endoproteinase LysC (Promega) and digested overnight at 37 °C. The reaction was stopped by addition of CF_3_COOH (TFA), and peptides were collected, vacuum dried and resuspended in the solution containing heavy-labeled peptide.

The samples were analyzed using a Q-Exactive mass spectrometer (Thermo Scientific) coupled with nano-HPLC (UltiMate 3000 RSLCnano System, Thermo Scientific). Peptides were loaded onto a trap column (AcclaimPepMap100 C18, Thermo Scientific; ID 75 μm, length 20 mm, particle size 3 μm, pore size 100 Å) in 2% ACN/0.05% TFA at a flow rate of 5 μl/min and then separated on an analytical column (AcclaimPepMapRLSC C18, Thermo Scientific; ID 75 μm, length 500 mm, particle size 2 μm, pore size 100 Å) using a 30 min gradient of ACN from 2 to 40% in the presence of 0.05% formic acid at a flow rate of 300 nl/min. A digital PicoView 550 ion source (New Objective) was used for ionization. Labeled peptide and its light counterpart were isolated in scheduled PRM with a 2 m/z window and fragmented with a normalized collision energy of 25. Resulting ions were collected with a maximum injection time of 500 ms and an automatic gain control (AGC) target value of 2.0 × 10^4^. Measurements were collected at a resolution of 140,000. Data were analyzed using Skyline software (version 3.7). The signal corresponding to the SPSPSPTSPGSLRK peptide was verified by confirming its coelution with the heavy-labeled counterpart and by comparing the fragment ion area ratios in peptide pairs.

## Results

### Changes in *Dclk1* expression in response to psychotropic drugs

**Fig. 1:**
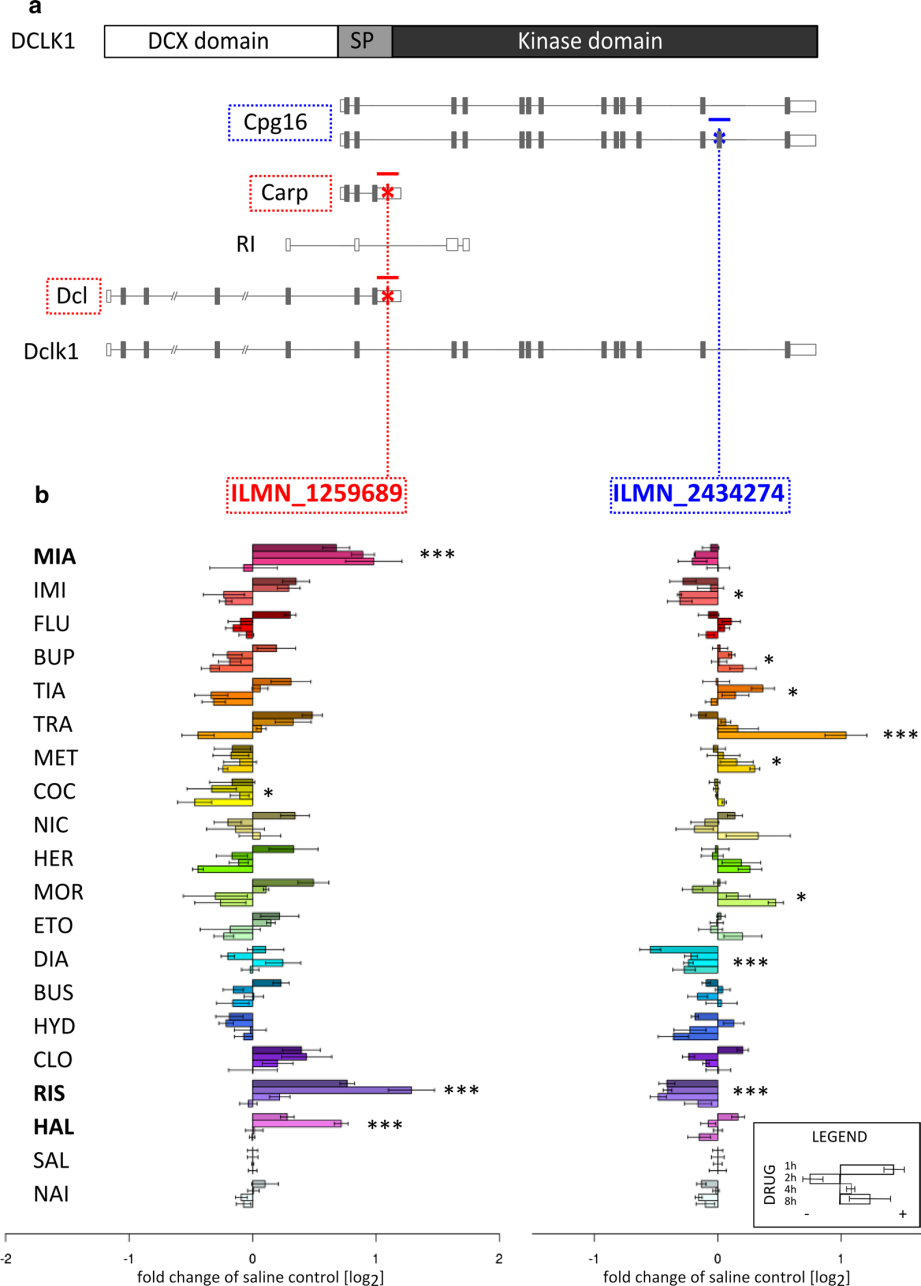
Schematic representation of the *Dclk1* gene and psychotropic drug treatment-mediated regulation of its expression. **a** Main transcripts of the *Dclk1* locus: two Cpg16 variants (ENSMUST00000198437, ENSMUST00000070418), Carp (ENSMUST00000199585), intron-retained isoform (RI, ENSMUST00000198757), Dcl (ENSMUST00000167204) and Dclk1 (ENSMUST00000054237). The positions of the microarray probes are indicated by the stars. The schematic was generated based on information in the Ensembl database and available literature [6, 26]. The functional protein domains are boxed (SP is the serine/proline-rich domain). **b** Results from the microarray gene expression analysis are presented as time courses (1, 2, 4 and 8 h) of fold changes in expression compared to levels observed in saline-treated controls (as described in the legend). The measurements from two microarray probes are presented. The left side of the figure shows *Dclk1* levels based on the ILMN_1259689 probe, whereas the right side corresponds to ILMN_2434274. The list of analyzed drugs include mianserin (MIA), imipramine (IMI), fluoxetine (FLU), bupropion (BUP), tianeptine (TIA), tranylcypromine (TRA), methamphetamine (MET), cocaine (COC), nicotine (NIC), heroin (HER), morphine (MOR), ethanol (ETO), diazepam (DIA), buspirone (BUS), hydroxyzine (HYD), clozapine (CLO), risperidone (RIS), haloperidol (HAL), saline (SAL), and naive (NAI) control. *P < 0.01, **P < 0.001, ***P < 0.0001, one-way ANOVA of the drug factor

First, we reexamined the expression of alternatively spliced transcripts from the *Dclk1* locus based on the previously reported dataset describing the effects of various psychotropic drugs on the mouse striatum [2]. The results of the analysis are shown in Fig. 1. Panel A shows a schematic representation of the main transcripts of the mouse *Dclk1* gene based on the NCBI37/mm9 mouse genome release. Regions corresponding to probes from the MouseWG-6 v2 BeadChip are marked with blue or red symbols. Probes correspond to nonoverlapping transcripts and neither detected the full-length canonical *Dclk1* transcript. As shown in the reanalysis of the array data presented in panel B, levels of transcripts detected by the probes were differentially affected by treatments with psychotropic drugs.

The mRNA levels measured using the first microarray probe (ILMN_1259689, presented as a red star in Fig. 1; drug *P* value = 4.14 × 10^−16^, time P-value = 9.9 × 10^−15^, interaction P-value = 4.2 × 10^−5^) indicated increased expression after the administration of mianserin (1, 2 and 4 h after the injection), risperidone (1 and 2 h) and, to a lesser extent, haloperidol (2 h) treatments (Fig. 1b). The second probe (ILMN_2434274, blue star; drug P-value = 4.68 × 10^−16^, time P-value = 1.2 × 10^−4^, interaction P-value = 2.5 × 10^−7^) revealed a different pattern of regulation, with an increase in mRNA abundance levels 8 h after the tranylcypromine treatment. Analysis of the array profiling results showed isoform-specific regulation of *Dclk1* expression by psychotropic drugs. However, the interpretation was also confounded by ambiguous detection of the *Dclk1* transcripts.

### Transcriptome profiling of the effects of psychotropic drugs using RNA sequencing

We used next-generation sequencing to comprehensively examine drug-induced *Dclk1* gene expression at the level of specific transcriptional units. Sequencing was performed on ribo-depleted RNA samples derived from the mouse nucleus accumbens septi (NAc) and prefrontal cortex (PFCx) 2 h after treatment with antidepressants (venlafaxine and mianserin), antipsychotics (haloperidol and risperidone), a psychostimulant (methamphetamine) or a psychotomimetic (ketamine).

We measured normalized transcript abundance levels (FPKM) for all transcripts annotated in the GRCm38.p5 genome release using the Cufflinks package. A total of 110,327 different transcripts corresponding to 45,935 annotated genes were detected in the PFCx or NAc at the threshold of a mean FPKM ≥ 0.1.

The overall differences in drug-induced gene expression between the NAc and PFCx were assessed using a one-way ANOVA for drug factor performed separately in each tissue. We found 90 transcripts regulated by the treatment only in the NAc and 246 transcripts altered in the PFC (at FDR < 0.0001). The examples of regulated genes are *Map6* and *Cdkn1a* in the NAc, and *Bhlhe40* and *Fkbp5* in the PFC. We also identified 26 transcripts regulated in both the analyzed tissues above the threshold, including *Homer1*, *Sgk1* and *Fosb* (Additional file 1: Figure S1).

Drug-regulated changes in transcript levels were classified by biotypes and alternatively spliced events. Notably, 78% of regulated transcripts in the NAc and 65% of the transcripts in the PFCx encoded proteins, compared with 56% of the transcripts expressed in the NAc or PFCx under basal conditions (Additional file 2: Figure S2). Among the drug-induced alterations, an enrichment of protein-coding variants was observed, although the majority of noncoding, regulated transcripts were classified as intron-retained transcripts.

**Fig. 2:**
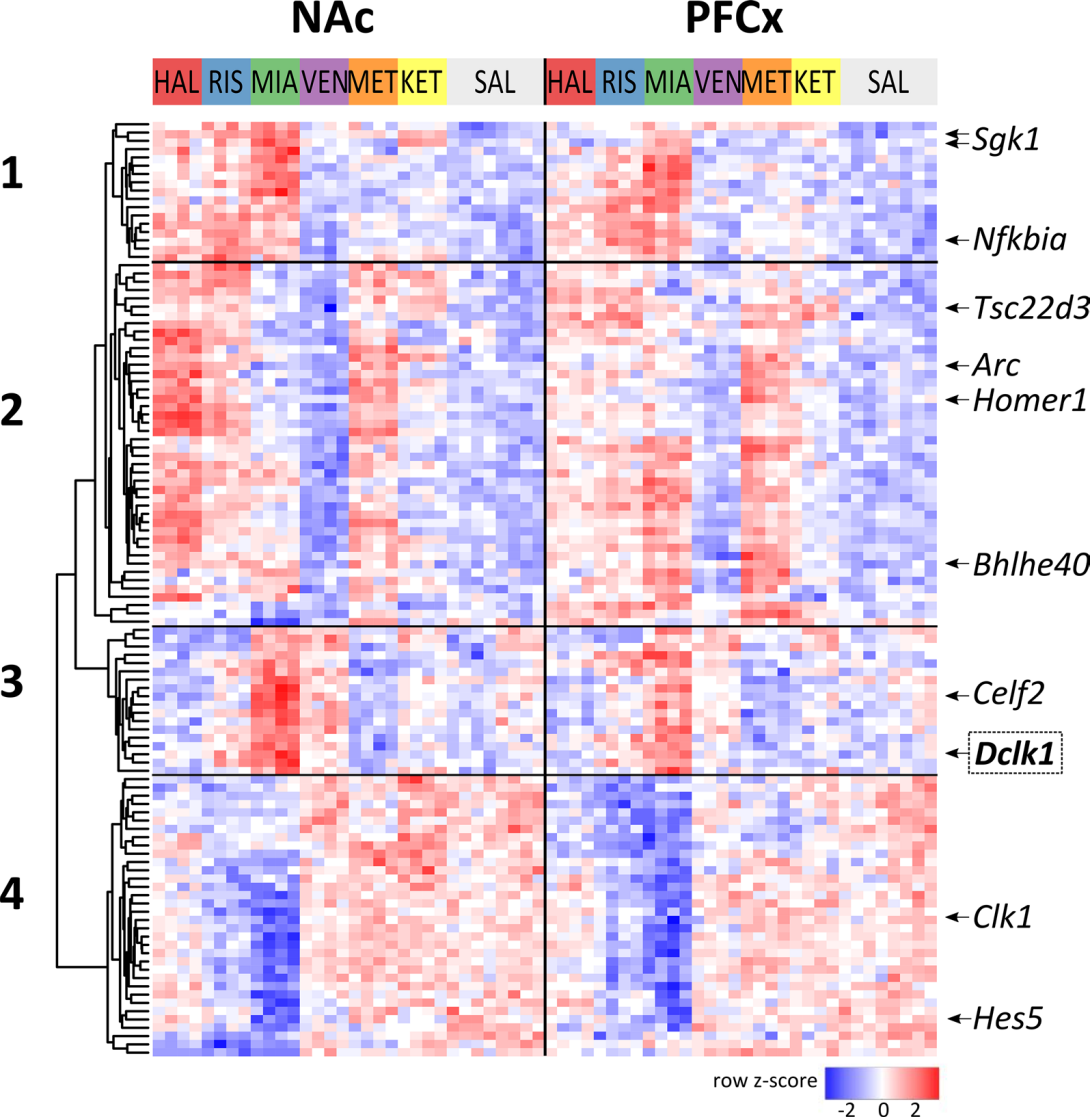
Drug-induced alterations in gene expression in the mouse NAc and PFCx. Hierarchical clustering analysis of drug-induced changes in gene transcript levels. RNA-seq results are shown as a heat map and include 113 transcripts with a genome-wide significance (FDR < 0.001 two-way ANOVA of the treatment factor). Transcriptional events regulated by the drugs are listed in Additional file 3. Colored rectangles represent the transcript abundance 2 h after the injection of the drug indicated above the rectangle (HAL: haloperidol; RIS: risperidone; MIA: mianserin; VEN: venlafaxine; MET: methamphetamine; KET: ketamine and SAL: saline control). Representative genes are presented on the right. The intensity of the color is proportional to the standardized value (z-score between − 2 and 2) of each RNA-seq measurement, as indicated on the bar below the heat map. Clustering was performed using Euclidean distances. Major branches from the clusters of drug-responsive changes are labeled 1–4

We used a two-way ANOVA with the drug and tissue as factors to identify drug-regulated changes in transcript levels (arbitrary cutoff at treatment effect FDR < 0.001; Additional file 3: Figure S3). Figure 2 shows the hierarchical clustering of the top 113 transcripts, grouped into four clusters. Transcripts from the four main branches were examined for overrepresented putative transcription factor binding sites in their promoter regions using seqinspector [20].

Transcripts from the first pattern were upregulated by mianserin in both brain regions. Their expression also increased in the NAc in response to risperidone and haloperidol treatments. Examples of transcripts clustered in this group included *Sgk1*, *Nfkbia* and the long noncoding RNA *Neat1*. Promoters of these transcripts exhibit a significant overrepresentation of the ChIP-seq signal for several transcriptional factors, including GR (P = 9.7 × 10^−6^, *t*-test with Bonferroni’s correction, track GEO accession: GSM686976), E2F1 (P = 1.1 × 10^−6^, GSM881056) and NFKB1 (P = 1 × 10^−5^, GSM88115). Haloperidol, methamphetamine and, to a lesser extent, risperidone induced the expression of the greatest number of transcripts (pattern 2) in the NAc. The strongest induction in the PFCx was observed after methamphetamine treatment. Pattern 2 included several genes involved in the molecular control of neuronal plasticity, such as *Fosb*, *Arc*, *Junb* or *Homer1*. The promoters of these transcripts contained a different set of putative transcriptional regulator binding sites, including SRF (P = 1.2 × 10^−22^, GSM530190) and EGR2 (P = 4.7 × 10^−12^, GSM881094). The expression of the third group of transcripts (pattern 3, e.g., *Celf2* and *Dclk1*) was induced by mianserin, with stronger effects observed in the NAc. We only identified one overrepresented potential transcriptional regulator of these genes, TBP (P = 3.5 × 10^−7^). The TBP binding motif was present in 18 of the 28 total genes. The *Dclk1* transcript was clustered into pattern 3. Notably, at the statistical threshold used to analyze the whole transcriptome, we observed significant changes in the levels of the transcript for only one *Dclk1* isoform, a noncoding variant with a retained intron (ENSMUST00000198757). Finally, the expression of transcripts from pattern 4, including *Clk1* and *Hes5*, decreased after mianserin treatment in both brain regions. Significantly overrepresented TFB sites were not present in the upstream promoter regions of transcripts from this cluster.

The profile of the drug-specific transcripts corresponding to the *Dclk1* locus was obtained by comparing the results of two high-throughput gene profiling methods-microarray (Fig. 1) and RNA-sequencing (Fig. 2).

### Isoform-specific regulation of *Dclk1* expression

**Fig. 3:**
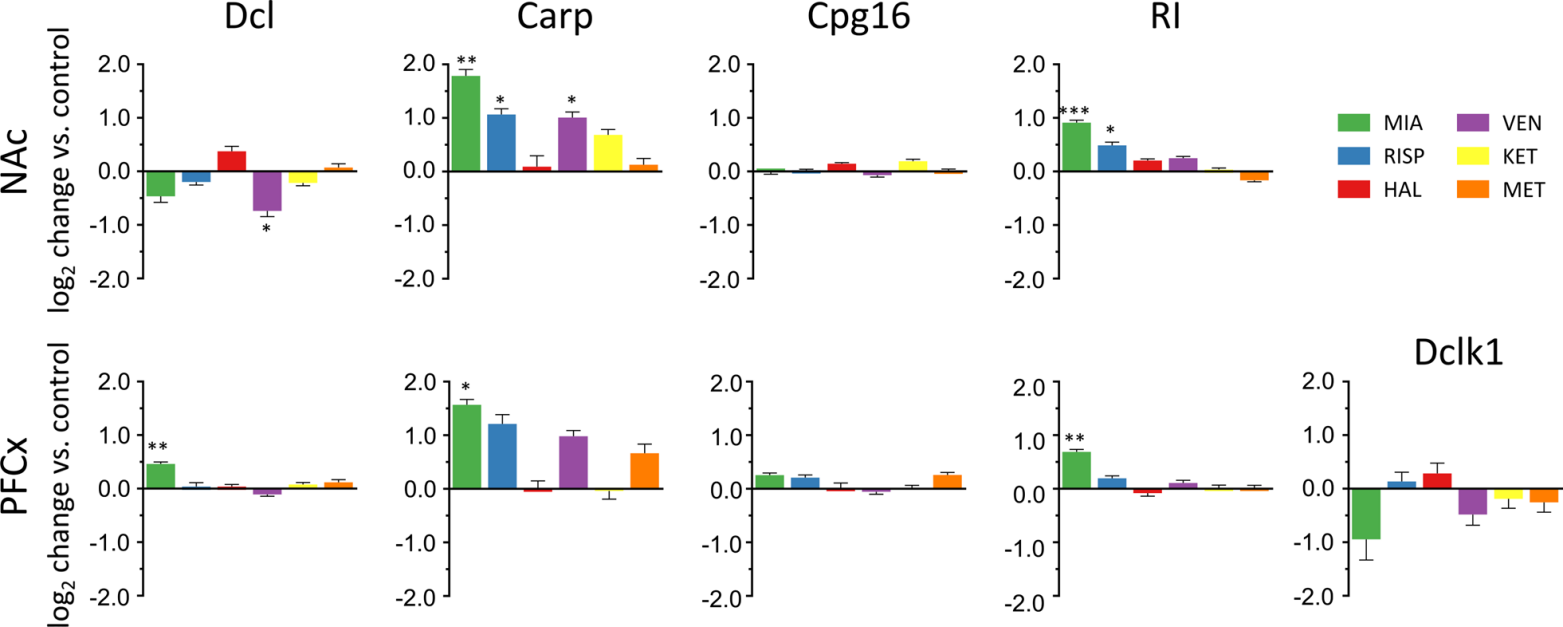
RNA-seq–based changes in levels of *Dclk1* transcripts. Bar graphs show log_2_-fold changes compared with those of the saline control. Bars indicate the S.E.M., *P < 0.01, **P < 0.001, ***P < 0.0001, one-way ANOVA of the drug factor

Next, we performed a detailed analysis of the sequencing results mapped to the *Dclk*1 locus. Based on the assignments from Cufflinks using the GRCm38.p5 mouse genome release, sequencing reads corresponded to 12 transcripts expressed from the *Dclk1* locus (at FPKM > 1), with 8 highly abundant isoforms (FPKM > 5). Three isoforms were not detected in the NAc or PFCx (ENSMUST00000198821, ENSMUST00000197870, and ENSMUST00000196745). The majority of the sequencing reads corresponded to Carp (ENSMUST00000199585), Dcl (ENSMUST00000167204) and Cpg16 (ENSMUST00000198437). Two of the transcripts assembled by Cufflinks contained retained introns and had not previously been described (Additional file 4: Figure S4). Relative changes in the abundance of the transcripts are shown in Fig. 3.

The expression of the Carp transcript in the NAc was induced in response to mianserin (log_2_FC = 1.78, P = 0.0002), risperidone (log_2_FC = 1.07, P = 0.007) and venlafaxine (log_2_FC = 1.01, P = 0.009). Carp expression in the PFCx was only regulated only mianserin (log_2_FC = 1.57, P = 0.0027). The level of the Cpg16 transcript was not significantly altered in any of the analyzed brain regions upon drug treatment. The venlafaxine treatment downregulated the expression of the Dcl isoform in the NAc (log_2_FC = − 0.74, P = 0.078). However, the level of the Dcl transcript in the PFCx increased in response to the mianserin treatment (log_2_FC = 0.46, P = 0.0009). The expression of the full-length *Dclk1* transcript (ENSMUST00000054237) was below the level of detection in the NAc, and no significant changes were observed in the PFCx. The expression of the intron-retained isoform (RI) was substantially upregulated by mianserin (log_2_FC = 0.9, P = 2.8 × 10^−6^) and risperidone (log_2_FC = 0.49, P = 0.002) in the NAc and by mianserin (log_2_FC = 0.69, P = 0.0003) in the PFCx.

**Fig. 4:**
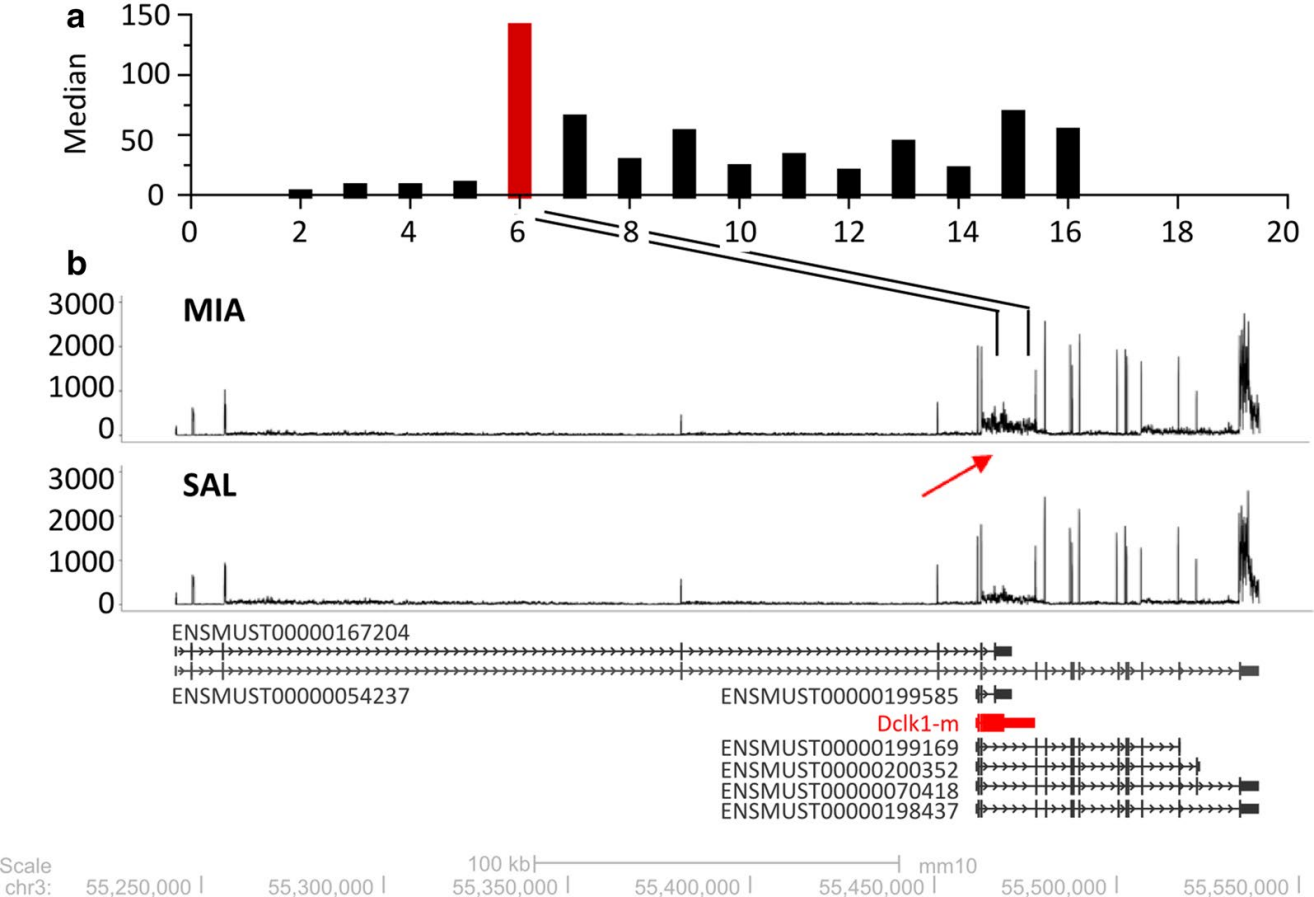
Identification of novel drug-regulated isoform of *Dclk1*. **a** Transcript levels measured as the median read coverage from each particular intronic region. The signal from the consecutive *Dclk1* introns are presented on the x-axis. **b** Representative RNA-seq tracks showing the transcriptional profile of the Dclk1 locus. The transcripts are annotated based on the Ensembl database, as well as the novel drug-regulated isoform Dclk1-m and are presented below. The arrow indicates drug-induced regulation of the transcript level

Furthermore, when we mapped individual reads to the *Dclk1* locus, a considerable number of fragments did not match known transcripts and corresponded to a fragment annotated as an intronic region (Fig. 4b, red arrow). Exon-level analysis of the data suggested the presence of a novel transcript, referred to as Dclk1-m, with an alternative transcription start site (first exon), termination codon (last exon) and a sequence corresponding to the sixth intron of the canonical *Dclk1* transcript (ENSMUST00000054237). The detected retention of intron 6 is unlikely to correspond to an unspliced pre-mRNA. The level of intron coverage calculated as the median read coverage for each position at each intron was significantly higher (P = 0.002) for this intron compared with that of other introns (Fig. 4a).

**Fig. 5:**
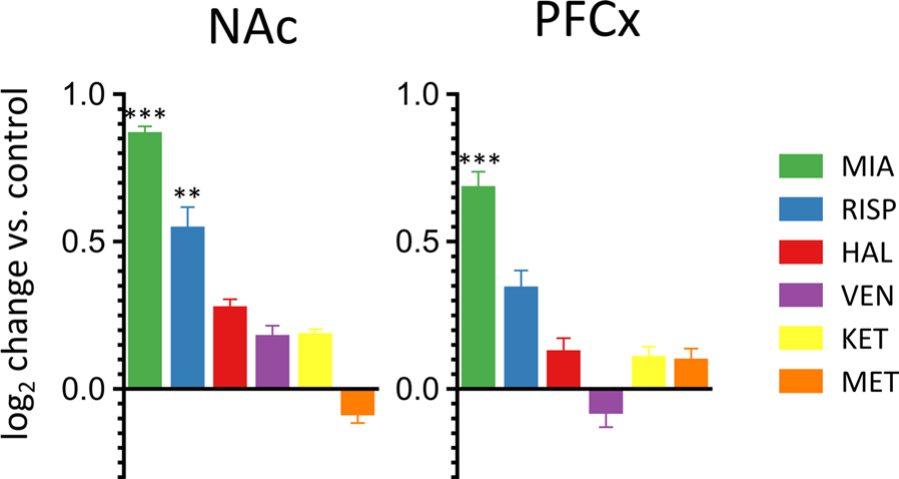
Drug-induced changes in the expression of a novel *Dclk1* isoform (Dclk1-m). Changes in the level of Dclk1-m measured by RNA-seq. The results are shown as log_2_-fold changes compared with those of the saline control. Bars indicate S.E.Ms, **P < 0.001, ***P < 0.0001, one-way ANOVA of the drug factor

Based on the frequency of the sequence reads corresponding to the Dclk1-m variant, the expression of this isoform was upregulated in the PFCx by the mianserin treatment (log_2_FC = 0.679, P = 0.0000253) and in the NAc by mianserin and risperidone (log_2_FC = 0.862, P = 3.8 × 10^−8^; log_2_FC = 0.542, P = 0.00079, respectively; Fig. 5 and Additional file 5: Figure S5). Notably, the ILMN_1259689 probe shown in Fig. 1 hybridized with Dclk1-m. In conclusion, next-generation sequencing analysis confirmed the drug-specific induction of transcription from the Dclk1 locus and showed that psychotropic drugs affected the transcription of a short region close to the sequence encoding the serine-proline rich domain. Most importantly, changes in transcription might actually represent a novel transcript, Dclk1-m.

### Validation of drug-induced expression of *Dclk1* isoforms

**Fig. 6:**
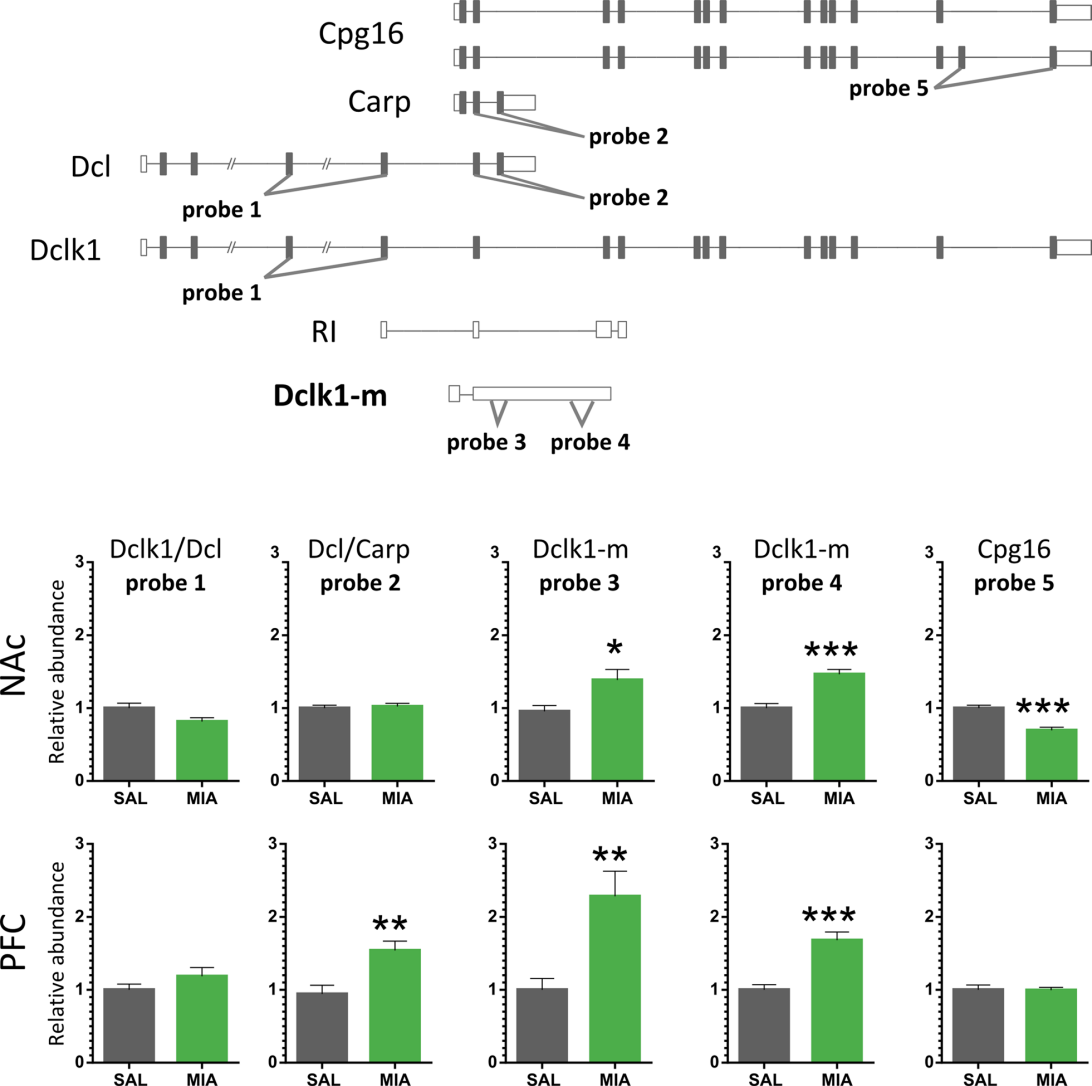
Validation of mianserin-induced changes in *Dclk1* expression. Changes in mRNA levels were measured 2 h after administration of mianserin or saline. qPCR analyses were performed using samples from an independent biological experiment (n = 8). TaqMan probes distinguished the following specific transcriptional variants: qPCR “probe 1” spans the exon junction of exons 4 and 5 of the Dclk1 (ENSMUST00000054237) transcript, qPCR “probe 2” spans exons 6 and 7 in Dcl (ENSMUST00000167204), qPCR “probe 3” spans 5′ part of intron 6 of Dclk1 (ENSMUST00000054237), and qPCR “probe 4” spans 3′ part of intron 6 of Dclk1 (ENSMUST00000054237), qPCR “probe 5” spans exons 13 and 14 of Cpg16 (ENSMUST00000198437). The locations of TaqMan probes used for qPCR are labeled (probes 1–5). Bars indicate the S.E.M, ***P < 0.001, one-way ANOVA of the drug factor

We designed a series of isoform-specific probes for qPCR to confirm which of the transcripts exhibited increased expression in response to drug treatments. The analysis was performed on a new set of samples obtained from mice euthanized 2 h after the i.p. injection of mianserin (20 mg/kg), risperidone (0.5 mg/kg), haloperidol (1 mg/kg) or saline (n = 8). Five fluorescent probe assays were used to analyze changes in the expression of *Dclk1* transcripts; two of the assays were specifically designed to detect the isoform containing the retained intron (Fig. 6, 5′ region of intron 6 and 3′ region of intron 6).

The mianserin treatment increased the levels of transcripts containing intron 6 in the NAc (approximately 1.5-fold). The level of Cpg16 was slightly decreased, whereas levels of Dcl/Carp were not different from those of the saline-treated control group. Risperidone significantly induced the expression of the Dclk1/Dcl isoform (approximately 1.2-fold), as well as transcripts containing intron 6 (approximately 1.6- to 2.1-fold). The haloperidol treatment had no effect on the expression of *Dclk1* isoforms in the NAc. In the PFCx, the mianserin treatment upregulated the expression of Dcl/Carp isoforms (1.5-fold), as well as transcripts containing intron 6 (more than twofold). Risperidone did not regulate the expression of *Dclk1* isoforms in the PFCx. The haloperidol treatment slightly decreased the expression of a variant containing part of intron 6 in the PFCx (Fig. 6 and Additional file 6: Figure S6).

We also performed two additional experiments and measured the transcript levels 4 h after a single treatment and 2 h after 5 days of treatment. The mianserin treatment significantly increased levels of Dclk1-m in the NAc. The effect of mianserin on *Dclk1* expression was not detected 5 days after repeated drug treatment (Additional file 7: Figure S7). Together, these data confirm that Dclk1-m expression is acutely increased in response to the mianserin treatment. Levels of other *Dclk1* transcripts were not increased in response to the mianserin treatment, with the possible exception of Carp.

### Functional features of the newly detected *Dclk1* variant

**Fig. 7:**
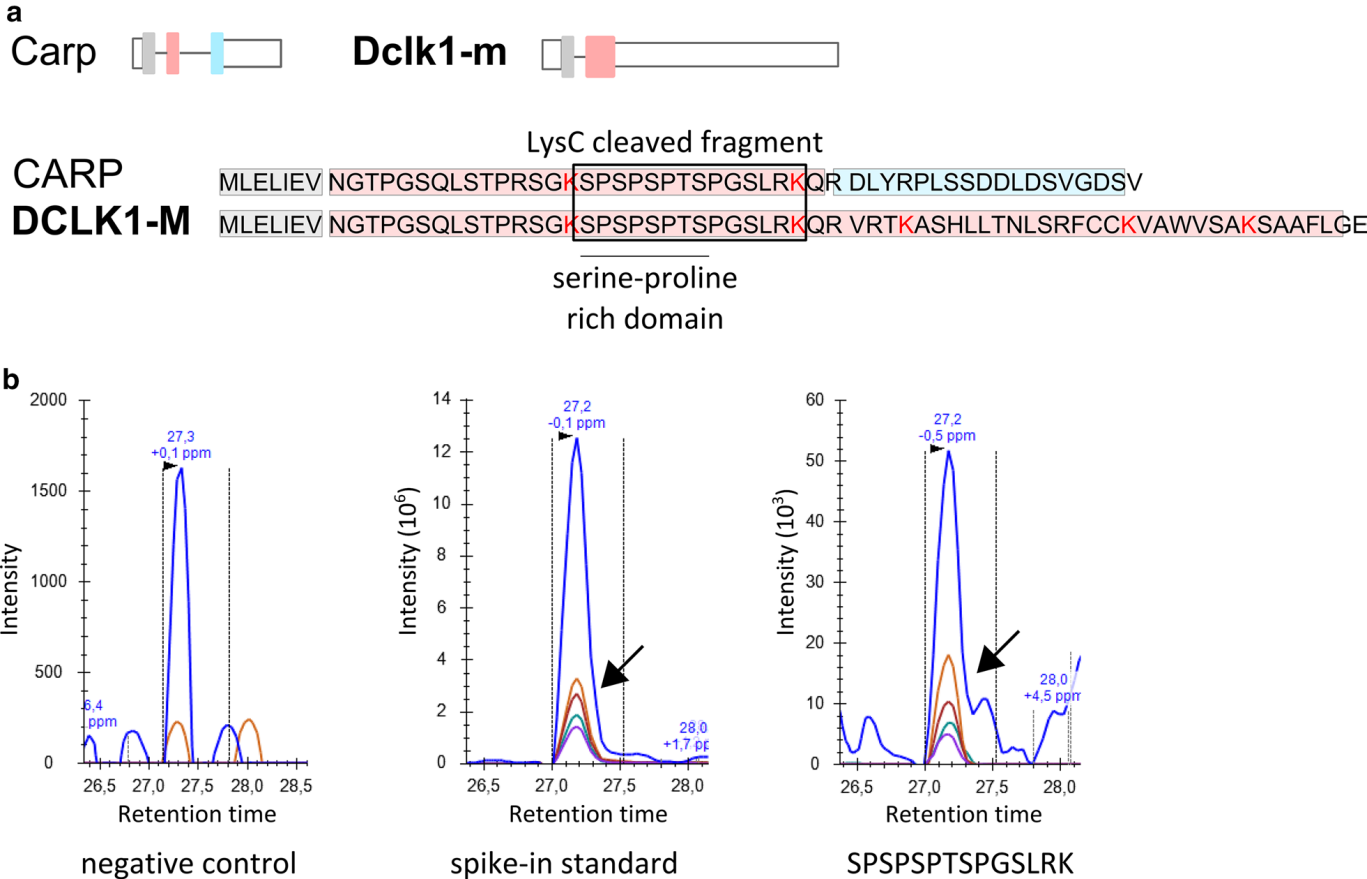
LC–MS/MS detection of CARP/DCLK1-M-derived peptide. **a** A schematic representation of the Carp and Dclk1-m transcripts and their putative peptide products. Colored boxes mark exons. Peptides were digested with the endoproteinase LysC, which cleaves peptide bonds at the carboxyl side of lysine residues (marked red). The fragment corresponding to the SPSPSPTSPGSLRK peptide is marked with a box. **b** Detection of the SPSPSPTSPGSLRK peptide. The panels show (from the left): signal for potential contamination with light peptide checked in heavy peptide sample, signal for heavy-labeled peptide spiked into the experimental sample, signal for endogenous (light) peptide in a sample derived from the murine striatum. Colored peaks correspond to peptide fragment ions. Please note the different scales on the y-axes

Finally, we used mass spectrometry to determine whether protein products of the Dclk1-m transcript were detected. Dclk1-m and Carp sequences overlap on the 5′ side of the putative protein-coding sequences; only the C-termini differ. Both sequences include the proline- and serine-rich (SP-rich) region that interacts with other proteins. Mass spectrometry analysis of the SDS-PAGE-purified protein fraction with a molecular weight less than 10 kDa confirmed the presence of the SP-rich fragment (Fig. 7). Shotgun LC–MS/MS analysis, performed in addition to PRM, did not show a presence of other fragments derived from either CARP or DCLK1-M. Thus, we were only able to conclude that either or both the DCLK1-M and CARP proteins are translated.

## Discussion

Our results reveal the existence of a novel *Dclk1* isoform (Dclk1-m), the expression of which is induced in the nucleus accumbens and prefrontal cortex of the brain after treatment with psychotropic drugs that affect 5HT2A/C receptor signaling (i.e., mianserin and risperidone). It should be noted here that these drug-related effects are relatively unspecific in pharmacological action, and transcription might be triggered by the activity of more than one type of neuronal receptors. Notably, Dclk1-m is the main, if not exclusive *Dclk1* transcript whose expression appears to be regulated by drugs. At present, we were not able to unequivocally establish whether Dclk1-m is translated; we were only able to confirm that protein products of Dclk1-m or Carp were present.

The identification of Dclk1-m revises and extends observations from our previous reports, where we detected an increase in *Dclk1* expression after treatment with mianserin and risperidone [2, 3, 13]. However, due to limitations regarding the array methodology used in our previous study, we were not able to discern exactly which transcripts were induced by drug actions in many cases. The array probes used in gene expression arrays may actually be detecting Dclk1-m, Carp and Dcl. Furthermore, we postulate that other previous reports may have been reporting changes in Dclk1-m expression, as well. We concluded that drug treatment regulated the expression of brain-specific isoforms of *Dclk1* with an alternatively spliced last exon—either Dcl or Carp. In-depth analysis of RNA-seq data revealed that psychotropic drug treatments induced the expression of a previously unannotated transcript. The expression of this transcript was upregulated in the NAc by mianserin and risperidone and in the PFCx by mianserin. The Dclk1-m isoform contains a retained intron covering the middle part of the *Dclk1* locus. Thus, this intron is a region of the gene important for the mechanisms of action of those drugs. Both mianserin and risperidone are potent antagonists of 5-HT2a receptors, which might mediate the effect on Dclk1-m transcription. Moreover, the addition of mianserin to typical antipsychotics improves their therapeutic efficacy in patients with schizophrenia [22]. Polymorphisms in the *Dclk1* gene are associated with the development of schizophrenia. Thus, the altered function of the *Dclk1* gene may be involved in both the etiology and treatment of this psychopathology. From our results, we can’t ascertain whether the increase in Dclk1 isoform is sufficient to produce the therapeutic effects of the drugs. It is likely a more complex phenomenon and no single gene or transcript is solely responsible for the therapeutic effect.

The *Dclk1* gene has a complex structure and comprises at least 15 known transcriptional variants. Although *Dclk1* was first described as a brain-specific gene, the roles of *Dclk1* outside the nervous system have recently been the main area of study. *Dclk1* is expressed in a variety of cancer cells [23], with a functional role in tumor growth and progression. However, its general role in carcinogenesis and clinical significance as a potential diagnostic marker remain unclear [24]. The present analysis reveals the brain-region-specific regulation of the expression of *Dclk1* isoforms in response to treatments with various classes of psychotropic drugs. A comparative meta-analysis of drug-induced *Dclk1* expression profiles is difficult to perform due to the use of different experimental designs (organism, time and tissue selection) and methodologies. To our knowledge, this study is the first to show that *Dclk1* expression is regulated at the single-exon level. In general, our results are consistent with previous findings and complement them in many ways. The short, the *Dclk1* transcript Carp (also known as Ania-4) is considered an immediate early gene (IEG). The expression of this variant increases in striatal neurons within minutes of dopamine receptor D1 (D1) stimulation [25]. In the hippocampus, the Carp mRNA was upregulated following kainate-elicited seizures [9], adrenalectomy [26], brain-derived neurotrophic factor-induced long-term potentiation [27] and repeated exposures to glutamate in hippocampal slice cultures [28]. We only identified one study showing the regulation of *Dclk1* expression by psychotropic drugs; this study reported that chronic haloperidol, but not clozapine or olanzapine treatments [29], affect *Dclk1* transcription. However, based on the reported results, we were not able to distinguish between Dcl, Carp and Dclk1-m. Based on our results, the upregulation of Dclk1-m expression is transient, likely lasting only hours, and was not detected after repeated treatment with mianserin.

Interestingly, Dclk1-m potentially encodes a protein that includes the serine- and protein-rich peptide. Using mass spectrometry, we confirmed the presence of small proteins containing the SP-rich domain that were potentially derived from CARP or DCLK1-M. Therefore, the newly identified variant might be translated into a protein. The SP-rich domain shared by the CARP and DLCK1-M peptides is postulated to be responsible for interactions with other proteins. To our knowledge, the endogenous CARP peptide has never been identified in vivo. This domain is predicted to interact with proteins containing the Src homology (SH3) domain [30]. Indeed, CARP interacts with the adapter protein growth factor receptor-bound 2 (Grb2) that contains the SH3 domain in vitro [26]. Grb2 plays a role in the formation of dendritic spines, which is critical for synaptic development [31] and is implicated in regulating actin cytoskeleton dynamics [32] and in Ras-ERK kinase activation [33]. Thus, the observed effects on Dclk1-m expression may correspond to alterations at the protein level. We were not able to unequivocally establish whether Dclk1-m is translated, although we did confirm that protein products translated from Dclk1-m or Carp were indeed present.

Recent results suggest the involvement of DCLK1 in dendrite development, as the depletion of endogenous DCLK1 affects the complexity of dendritic branching and total dendritic length in neurons in vitro [34]. DCLK1 plays a critical role in cargo transport into dendrites; in particular, DCX domains are required for dense-core vesicle (DCV) trafficking that causes dendritic growth through the release of peptide neuromodulators [35]. Notably, DCLK1 kinase activity is not required for its ability to bind and bundle microtubules. One of the C-terminal variants (Cpg16) has also been suggested to be a candidate neural plasticity gene with a potential role in synaptic remodeling. In vitro, CPG16 autophosphorylates and phosphorylates myelin basic protein, but the in vivo target of CPG16 remains unknown. CPG16 may be activated by a PKA-induced pathway. CARP was proposed to both modulate kinase activity [9] and enhance DCL-induced tubulin polymerization [36] in vitro. The proposed roles in synaptic plasticity were based in large part on in silico structural analysis. Taken together, the effects of the drug-induced expression of Dclk1-m on controlling synaptic dynamics might be mediated at multiple molecular levels.

Another interesting observation from our study is the expression levels of specific *Dclk1* isoforms. Generally, isoforms driven by the upstream promoter are expressed at high levels in mice during early stages of life (P0–P5), whereas downstream promoter-derived transcripts are adult-specific [37]. In our study, the most abundant isoform in both the NAc and PFCx is Cpg16, which is consistent with the literature [6]. Carp was reported to be exclusively expressed in adulthood, although some reports show that its expression in the mouse brain is undetectable under basal conditions but dramatically increases upon stimulation [25]. One of the unexpected findings from our study is that the full-length *Dclk1* isoform was detected in PFCx, although it was reported to only be expressed during brain development [6, 36]. Dcl expression has been reported in specific neuronal cell populations and implicated in adult neurogenesis [38, 39]. We concluded that the expression level of *Dclk1* isoforms might exhibit brain region-specific patterns. The number and different proportions of *Dclk1* isoforms make it very difficult to study in vitro. We aim to evaluate the expression of *Dclk1* isoforms in primary cultures of neurons and astrocytes [3]. We observed significant differences in the levels of *Dclk1* isoforms between primary cultures and brain tissues. Notably, the *Cpg16* isoform was expressed at high levels in vivo but was virtually undetectable in cell cultures. The full-length *Dclk1* transcript was expressed at higher levels in cultured neurons than in brain tissues. This finding is not surprising because primary neuronal cultures are derived from mouse embryos. Moreover, stimulation of neurons with kainic acid (activity-regulated gene expression) and astrocytes with dexamethasone (GR-dependent gene expression) did not change the expression of any *Dclk1* isoforms. Thus, studies of the mechanisms regulating the expression of *Dclk1* isoforms in the brain should not be conducted in vitro.

## Conclusions

In conclusion, we identified a previously unknown *Dclk1* transcript, which is expressed in response to treatment with psychotropic drugs. This study enhances our understanding of brain plasticity by revealing that the expression of alternative *Dclk1* transcripts is an important component of the pharmacological treatment of neuropsychiatric disorders. Although further studies will allow researchers to determine the precise role of the newly discovered Dclk1-m variant, our findings reveal a previously unknown molecular mechanism of mianserin action. Moreover, our research also emphasizes the need to carefully analyze the raw data obtained from high-throughput gene expression profiling experiments, as analyses that are restricted to previously annotated transcripts might provide us with false-positive results, as in the case of *Dclk1* transcripts. The data reported here may also serve as a set of drug-specific transcriptional signatures in the PFCx and NAc.

## Additional files

**Additional file 1.** The lists of transcripts and corresponding gene names that are altered by drug treatment after one-way ANOVA in each tissue (FDR < 0.0001).

**Additional file 2.** The charts presenting the distribution of transcripts biotypes among the drug-regulated transcripts in comparison to the entire transcriptome of the mouse nucleus accumbens and prefrontal cortex.

**Additional file 3.** Results from two-way ANOVA for 113 drug-responsive transcripts (FDR for drug factor < 0.001) are included. For each transcript, a fold change over control, p-value and FDR for drug and tissue factors and all the interactions are presented. Table also includes biotypes annotation and classification using the BioMart interface to the Ensembl gene database.

**Additional file 4.** RNA-seq results from two-way ANOVA for all Dclk1 isoforms reported in Ensembl gene database (GRCm38.p5/mm10). For each transcript, a fold change over control, p-value and FDR for drug and tissue factors and all the interactions are presented.

**Additional file 5.** Table summarizing the RNA-seq re-analysis for Dclk1 locus with Dclk1-m variant added. The first sheet presents the results from one-way ANOVA for NAc, the second sheet for PFCx. For each transcript, a fold change over control, p-value and FDR for drug is presented.

**Additional file 6.** Changes in mRNA levels were measured 2 h after administration of risperidone, haloperidol or saline control. qPCR analyses were performed using samples from an independent biological experiment (n = 8). TaqMan probes distinguished the following specific transcriptional variants: qPCR “probe 1” spans the exon junction of exons 4 and 5 of the Dclk1 (ENSMUST00000054237) transcript, qPCR “probe 2” spans exons 6 and 7 in Dcl (ENSMUST00000167204), qPCR “probe 3” spans 5′ part of intron 6 of Dclk1 (ENSMUST00000054237), qPCR “probe 4” spans 3′ part of intron 6 of Dclk1 (ENSMUST00000054237), qPCR “probe 5” spans exons 13 and 14 of Cpg16 (ENSMUST00000198437). The locations of TaqMan probes used for qPCR are labeled in Figure 6. Bars indicate the S.E.M., *P < 0.01, one-way ANOVA of the drug factor.

**Additional file 7.** Changes in mRNA levels were measured (A) 4 h after administration of mianserin or saline control (B) 2 h after 5 days of treatment with mianserin or saline. qPCR analyses were performed using samples from an independent biological experiment (n = 4). The locations of TaqMan probes used for qPCR are labeled in Figure 6. Bars indicate the S.E.M., **P < 0.001, one-way ANOVA of the drug factor.

## Abbreviations

Dclk1: doublecortin-like kinase 1
FDR: false discovery rate
FPKM: fragments per kilobase of transcript per million fragments mapped
MS: mass spectrometry
NAc: nucleus accumbens
NGS: next-generation sequencing
PFCx: prefrontal cortex
PRM: parallel reaction monitoring

## References

[1] Brennand KJ, Simone A, Tran N, Gage FH (2012). Modeling psychiatric disorders at the cellular and network levels. Mol Psychiatry.

[2] Korostynski M, Piechota M, Dzbek J, Mlynarski W, Szklarczyk K, Ziolkowska B, Przewlocki R (2013). Novel drug-regulated transcriptional networks in brain reveal pharmacological properties of psychotropic drugs. BMC Genom.

[3] Piechota M, Golda S, Ficek J, Jantas D, Przewlocki R, Korostynski M (2015). Regulation of alternative gene transcription in the striatum in response to antidepressant drugs. Neuropharmacology.

[4] Gordon-Weeks PR, Fournier AE (2014). Neuronal cytoskeleton in synaptic plasticity and regeneration. J Neurochem.

[5] Shin E, Kashiwagi Y, Kuriu T, Iwasaki H, Tanaka T, Koizumi H, Gleeson JG, Okabe S (2013). Doublecortin-like kinase enhances dendritic remodelling and negatively regulates synapse maturation. Nat Commun.

[6] Burgess HA, Reiner O (2002). Alternative splice variants of doublecortin-like kinase are differentially expressed and have different kinase activities. J Biol Chem.

[7] Havik B, Degenhardt FA, Johansson S, Fernandes CP, Hinney A, Scherag A, Lybaek H, Djurovic S, Christoforou A, Ersland KM (2012). DCLK1 variants are associated across schizophrenia and attention deficit/hyperactivity disorder. PLoS ONE.

[8] Wu JQ, Wang X, Beveridge NJ, Tooney PA, Scott RJ, Carr VJ, Cairns MJ (2012). Transcriptome sequencing revealed significant alteration of cortical promoter usage and splicing in schizophrenia. PLoS ONE.

[9] Vreugdenhil E, Datson N, Engels B, de Jong J, van Koningsbruggen S, Schaaf M, de Kloet ER (1999). Kainate-elicited seizures induce mRNA encoding a CaMK-related peptide: a putative modulator of kinase activity in rat hippocampus. J Neurobiol.

[10] Burgess HA, Martinez S, Reiner O (1999). KIAA0369, doublecortin-like kinase, is expressed during brain development. J Neurosci Res.

[11] Silverman MA, Benard O, Jaaro H, Rattner A, Citri Y, Seger R (1999). CPG16, a novel protein serine/threonine kinase downstream of cAMP-dependent protein kinase. J Biol Chem.

[12] Engels BM, Schouten TG, van Dullemen J, Gosens I, Vreugdenhil E (2004). Functional differences between two DCLK splice variants. Brain Res Mol Brain Res.

[13] Ficek J, Zygmunt M, Piechota M, Hoinkis D, Rodriguez Parkitna J, Przewlocki R, Korostynski M (2016). Molecular profile of dissociative drug ketamine in relation to its rapid antidepressant action. BMC Genom.

[14] van de Waterbeemd H, Camenisch G, Folkers G, Chretien JR, Raevsky OA (1998). Estimation of blood–brain barrier crossing of drugs using molecular size and shape, and H-bonding descriptors. J Drug Target.

[15] Altamura AC, De Novellis F, Mauri MC, Gomeni R (1987). Plasma and brain pharmacokinetics of mianserin after single and multiple dosing in mice. Prog Neuropsychopharmacol Biol Psychiatry.

[16] Zetler G, Baumann GH (1985). Pharmacokinetics and effects of haloperidol in the isolated mouse. Pharmacology.

[17] Higashino K, Ago Y, Umehara M, Kita Y, Fujita K, Takuma K, Matsuda T (2014). Effects of acute and chronic administration of venlafaxine and desipramine on extracellular monoamine levels in the mouse prefrontal cortex and striatum. Eur J Pharmacol.

[18] Riviere GJ, Gentry WB, Owens SM (2000). Disposition of methamphetamine and its metabolite amphetamine in brain and other tissues in rats after intravenous administration. J Pharmacol Exp Ther.

[19] Paxinos G, Franklin KBJ (2001). The mouse brain in stereotaxic coordinates.

[20] Piechota M, Korostynski M, Ficek J, Tomski A, Przewlocki R (2016). Seqinspector: position-based navigation through the ChIP-seq data landscape to identify gene expression regulators. BMC Bioinform.

[21] Gabruk M, Nowakowska Z, Skupien-Rabian B, Kedracka-Krok S, Mysliwa-Kurdziel B, Kruk J (2016). Insight into the oligomeric structure of PORA from *A. thaliana*. Biochem Biophys Acta.

[22] Shiloh R, Zemishlany Z, Aizenberg D, Valevski A, Bodinger L, Munitz H, Weizman A (2002). Mianserin or placebo as adjuncts to typical antipsychotics in resistant schizophrenia. Int Clin Psychopharmacol.

[23] Shi W, Li F, Li S, Wang J, Wang Q, Yan X, Zhang Q, Chai L, Li M (2017). Increased DCLK1 correlates with the malignant status and poor outcome in malignant tumors: a meta-analysis. Oncotarget.

[24] Westphalen CB, Quante M, Wang TC (2017). Functional implication of Dclk1 and Dclk1-expressing cells in cancer. Small GTPases.

[25] Berke JD, Paletzki RF, Aronson GJ, Hyman SE, Gerfen CR (1998). A complex program of striatal gene expression induced by dopaminergic stimulation. J Neurosci Off J Soc Neurosci.

[26] Schenk GJ, Engels B, Zhang YP, Fitzsimons CP, Schouten T, Kruidering M, de Kloet ER, Vreugdenhil E (2007). A potential role for calcium/calmodulin-dependent protein kinase-related peptide in neuronal apoptosis: in vivo and in vitro evidence. Eur J Neurosci.

[27] Wibrand K, Messaoudi E, Havik B, Steenslid V, Lovlie R, Steen VM, Bramham CR (2006). Identification of genes co-upregulated with Arc during BDNF-induced long-term potentiation in adult rat dentate gyrus in vivo. Eur J Neurosci.

[28] Kawaai K, Tominaga-Yoshino K, Urakubo T, Taniguchi N, Kondoh Y, Tashiro H, Ogura A, Tashiro T (2010). Analysis of gene expression changes associated with long-lasting synaptic enhancement in hippocampal slice cultures after repetitive exposures to glutamate. J Neurosci Res.

[29] Duncan CE, Chetcuti AF, Schofield PR (2008). Coregulation of genes in the mouse brain following treatment with clozapine, haloperidol, or olanzapine implicates altered potassium channel subunit expression in the mechanism of antipsychotic drug action. Psychiatr Genet.

[30] Zarrinpar A, Bhattacharyya RP, Lim WA (2003). The structure and function of proline recognition domains. Sci STKE Signal Transduct Knowl Environ.

[31] Moeller ML, Shi Y, Reichardt LF, Ethell IM (2006). EphB receptors regulate dendritic spine morphogenesis through the recruitment/phosphorylation of focal adhesion kinase and RhoA activation. J Biol Chem.

[32] Buday L, Wunderlich L, Tamas P (2002). The Nck family of adapter proteins: regulators of actin cytoskeleton. Cell Signal.

[33] Katz ME, McCormick F (1997). Signal transduction from multiple Ras effectors. Curr Opin Genet Dev.

[34] Lipka J, Kapitein LC, Jaworski J, Hoogenraad CC (2016). Microtubule-binding protein doublecortin-like kinase 1 (DCLK1) guides kinesin-3-mediated cargo transport to dendrites. EMBO J.

[35] Lazo OM, Gonzalez A, Ascano M, Kuruvilla R, Couve A, Bronfman FC (2013). BDNF regulates Rab11-mediated recycling endosome dynamics to induce dendritic branching. J Neurosci Off J Soc Neurosci.

[36] Vreugdenhil E, Kolk SM, Boekhoorn K, Fitzsimons CP, Schaaf M, Schouten T, Sarabdjitsingh A, Sibug R, Lucassen PJ (2007). Doublecortin-like, a microtubule-associated protein expressed in radial glia, is crucial for neuronal precursor division and radial process stability. Eur J Neurosci.

[37] Pal S, Gupta R, Kim H, Wickramasinghe P, Baubet V, Showe LC, Dahmane N, Davuluri RV (2011). Alternative transcription exceeds alternative splicing in generating the transcriptome diversity of cerebellar development. Genome Res.

[38] Saaltink DJ, Havik B, Verissimo CS, Lucassen PJ, Vreugdenhil E (2012). Doublecortin and doublecortin-like are expressed in overlapping and non-overlapping neuronal cell population: implications for neurogenesis. J Comp Neurol.

[39] Kunze A, Achilles A, Keiner S, Witte OW, Redecker C (2015). Two distinct populations of doublecortin-positive cells in the perilesional zone of cortical infarcts. BMC Neurosci.

