# Supplementary figures and images for: Expression of alternatively spliced variants of the Dclk1 gene is regulated by psychotropic drugs
Source: BMC Neurosci. 2018 Sep 12;19:55. doi: 10.1186/s12868-018-0458-4 (PMC6134793; doi:10.1186/s12868-018-0458-4)

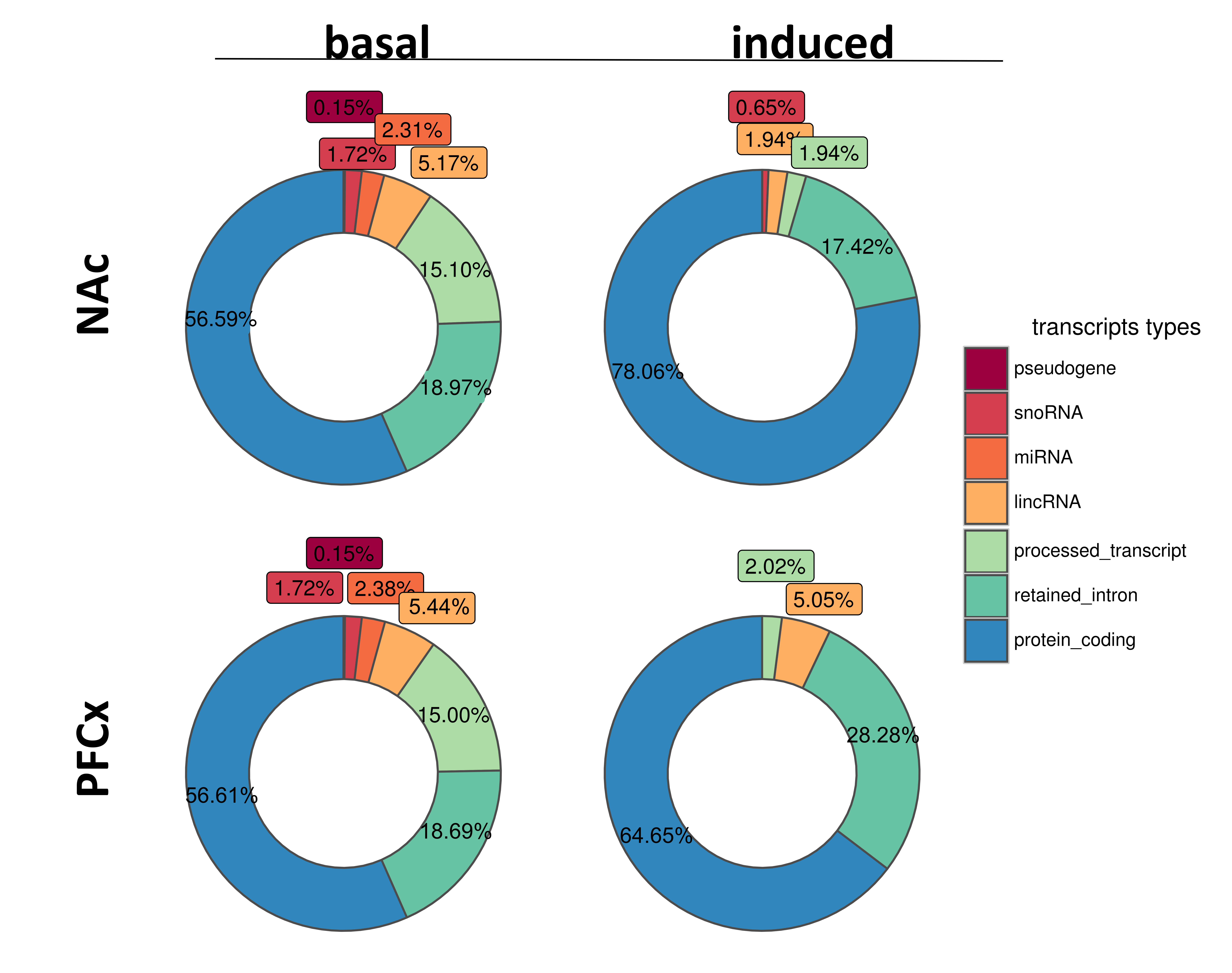

Supplement: Supplementary file 2 — Additional file 2. The charts presenting the distribution of transcripts biotypes among the drug-regulated transcripts in comparison to the entire transcriptome of the mouse nucleus accumbens and prefrontal cortex. [file 12868_2018_458_MOESM2_ESM.png]

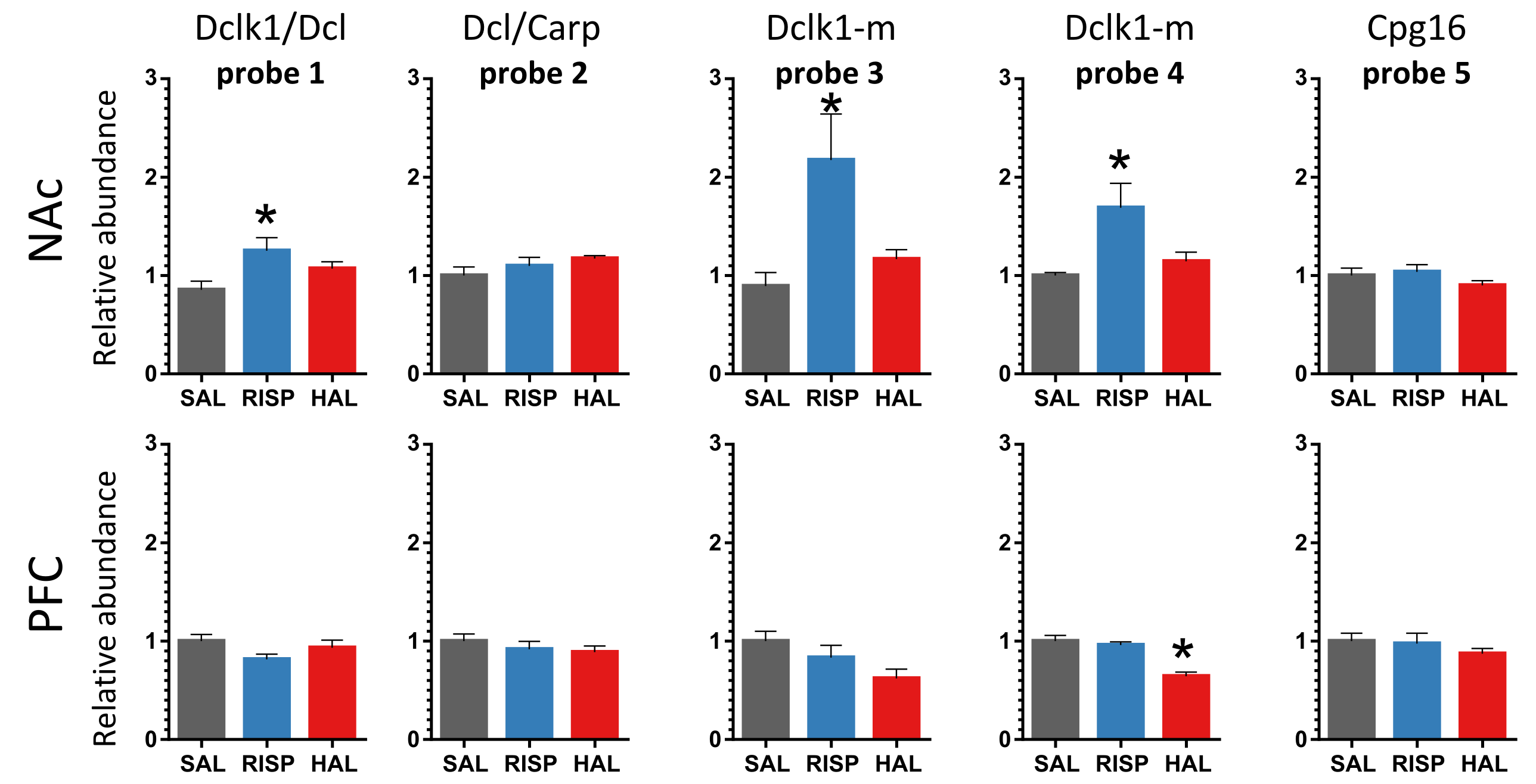

Supplement: Supplementary file 6 — Additional file 6. Changes in mRNA levels were measured 2 h after administration of risperidone, haloperidol or saline control. qPCR analyses were performed using samples from an independent biological experiment (n = 8). TaqMan probes distinguished the following specific transcriptional variants: qPCR “probe 1” spans the exon junction of exons 4 and 5 of the Dclk1 (ENSMUST00000054237) transcript, qPCR “probe 2” spans exons 6 and 7 in Dcl (ENSMUST00000167204), qPCR “probe 3” spans 5′ part of intron 6 of Dclk1 (ENSMUST00000054237), qPCR “probe 4” spans 3′ part of intron 6 of Dclk1 (ENSMUST00000054237), qPCR “probe 5” spans exons 13 and 14 of Cpg16 (ENSMUST00000198437). The locations of TaqMan probes used for qPCR are labeled in Figure 6. Bars indicate the S.E.M., *P < 0.01, one-way ANOVA of the drug factor. [file 12868_2018_458_MOESM6_ESM.png]

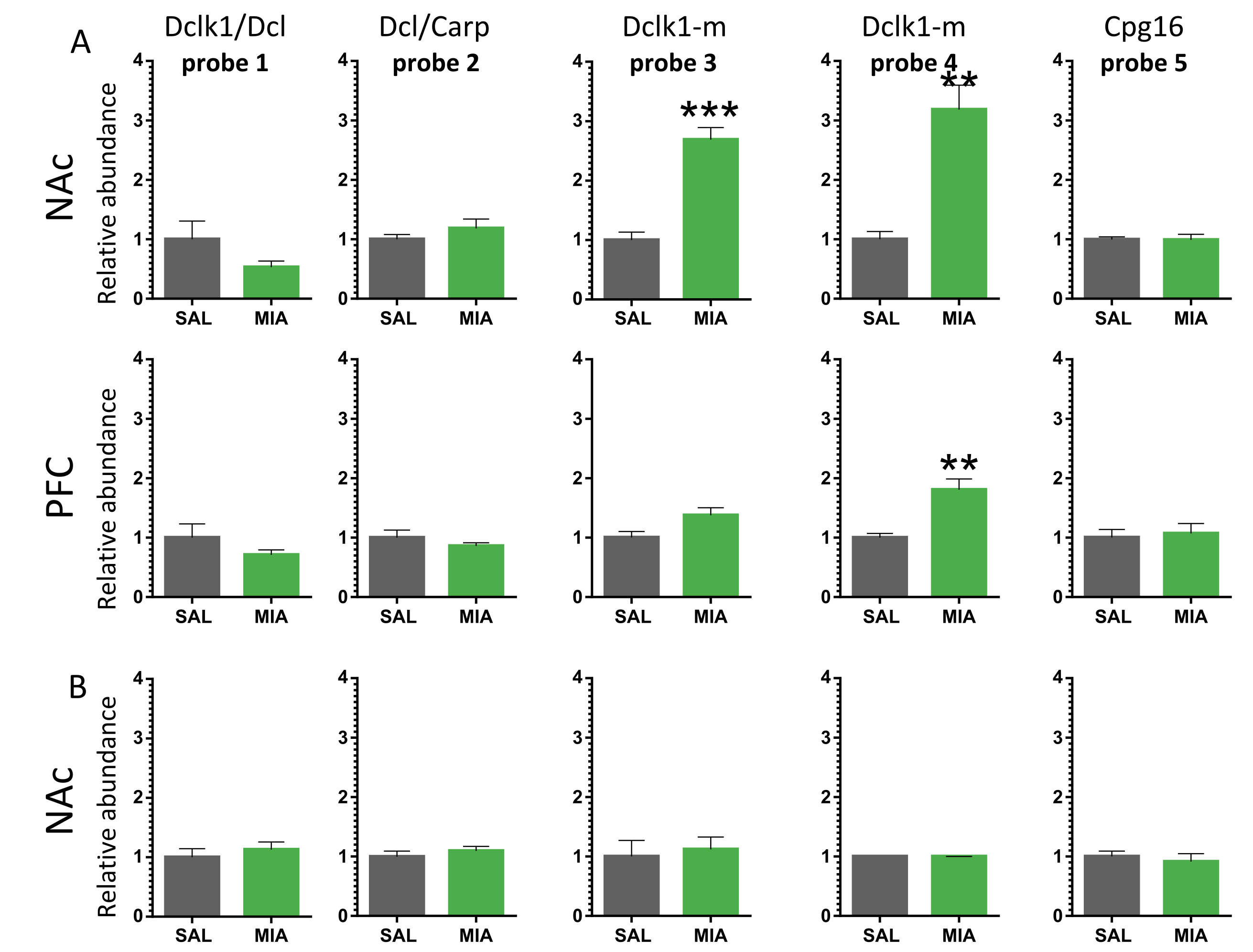

Supplement: Supplementary file 7 — Additional file 7. Changes in mRNA levels were measured (A) 4 h after administration of mianserin or saline control (B) 2 h after 5 days of treatment with mianserin or saline. qPCR analyses were performed using samples from an independent biological experiment (n = 4). The locations of TaqMan probes used for qPCR are labeled in Figure 6. Bars indicate the S.E.M., **P < 0.001, one-way ANOVA of the drug factor. [file 12868_2018_458_MOESM7_ESM.png]
